# Gangrenous Cystitis: A Rare Case Report

**DOI:** 10.7759/cureus.36233

**Published:** 2023-03-16

**Authors:** Christos N Noulas, Markos Markou, Rodopi Sotiropoulou, Dimitrios E Diamantidis, Michail Karanikas

**Affiliations:** 1 First General Surgery Department, University Hospital of Alexandroupolis, Democritus University of Thrace, Alexandroupolis, GRC

**Keywords:** emergency, laparotomy, urine retention, ischemia, bladder, gangrenous cystitis

## Abstract

Gangrenous cystitis is a rare condition of the urinary bladder with bladder wall ischemia as the main etiopathogenic factor and constitutes a surgical emergency. The risk factors for this condition include diabetes mellitus, prolonged labor, and topical chemotherapy, and the condition must be immediately treated because of its high mortality rate. This report describes a rare case of a patient with gangrenous cystitis who underwent radical surgical treatment; the incidence, etiology, diagnosis, management, and outcomes are also discussed.

## Introduction

Gangrenous cystitis is an uncommon condition, with only a few reported cases in the current literature [1]. Most reported cases were published a few decades ago when the use of antibiotics was not as prevalent as today. With the widespread use of antibiotics, gangrenous cystitis has become a rarely reported pathology [2].

In this report, the case of a 60-year-old male patient is presented who was first admitted to the internal medicine department because of fecal and urinary retention. After clinical deterioration, the patient was referred to the surgical department and diagnosed with gangrenous cystitis of unknown etiology. The patient underwent total prostatectomy, radical cystectomy, partial peritoneal excision, debridement of necrotic abdominal wall muscle tissues, and bilateral ureterostomy. Unfortunately, despite the radical treatment, the patient died on the third postoperative day owing to severe sepsis.

## Case presentation

A 60-year-old male patient presented to the emergency department complaining of diffuse abdominal pain with urinary retention and the absence of defecation for five days. His medical history included arterial hypertension and diabetes mellitus. Upon examination, the patient was found to be afebrile (body temperature 36.7℃) and hemodynamically stable (blood pressure: 105/60 mmHg, heart rate: 98 beats per min), with an oxygen saturation of 96%. Physical examination revealed profound abdominal distension, diffuse abdominal pain in all quadrants, and mild rebound tenderness.

Laboratory tests showed abnormal levels of white blood cells (15.27 K/µL with 93.3% neutrophils), serum glucose (684 mg/dL), urea (215 mg/dL), creatinine (2.1 mg/dL), Na+ (123 mmol/L), C-reactive protein (75.28 mg/dL), and lactate (5.5 mmol/L). Urine was dark brown and cloudy, and urinalysis showed a pH of 5.5; high specific gravity; and the presence of glucose, hemoglobulin, and protein (Table 1).

**Table 1:** Lab results on admission. Na+: sodium ion.

		Normal values
White blood count	15.27 K/µL	3.5-10.8 K/µL
Neutrophils	93.3%	40%-75%
Serum glucose	684 mg/dL	70-100 mg/dL
Urea	215 mg/dL	20-50 mg/dL
Creatinine	2.1 mg/dL	0.8-1.4 mg/dL
Na^+^	123 mmol/L	136-146 mmol/L
C-reactive protein	75.28 mg/dL	<1 mg/dL
Lactate	5.5 mmol/L	< 1.7 mmol/L
Urine pH	5.5	5-8
Urine specific gravity	>1030	1000-1030
Urine glucose	Positive	Negative
Urine hemoglobulin	Positive	Negative
Urine protein	>30 mg/dL	<30 mg/dL

**Figure 1:**
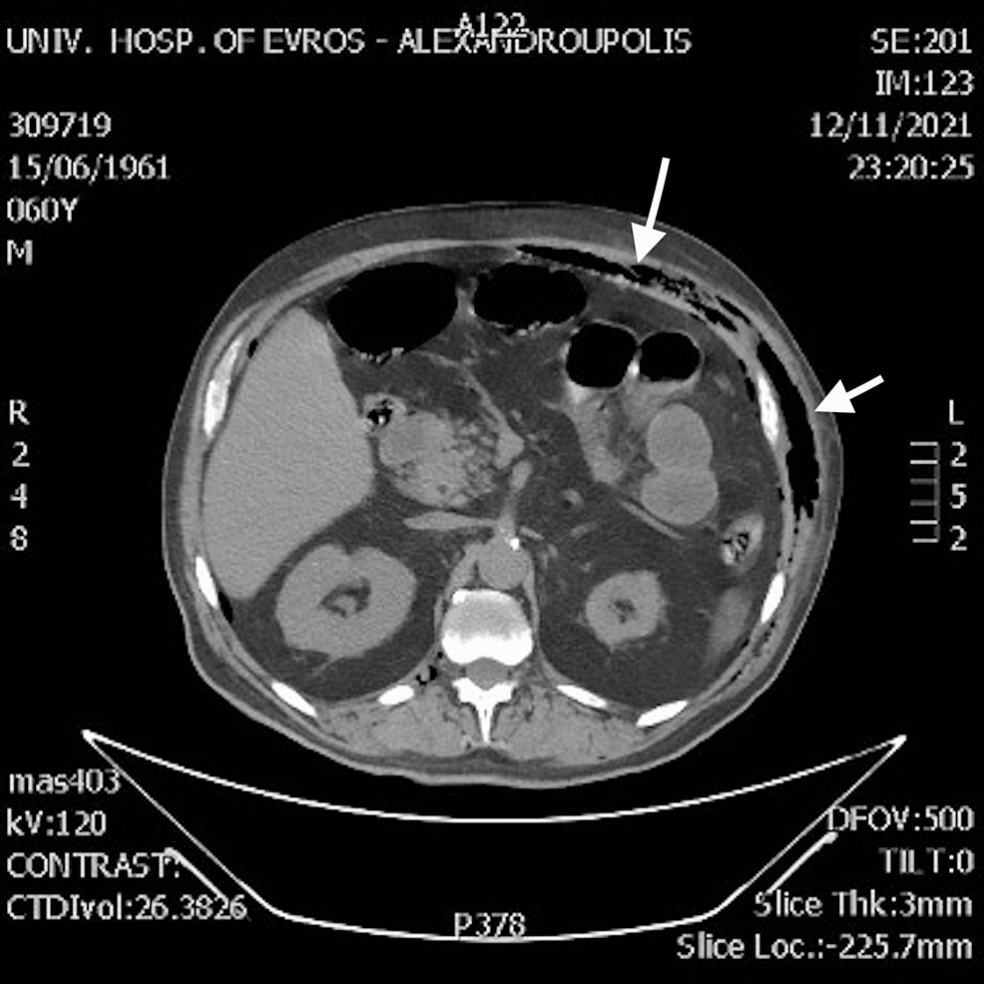
CT examination of the abdomen revealed free air in the abdominal wall (white arrows). CT: computed tomography.

**Figure 2:**
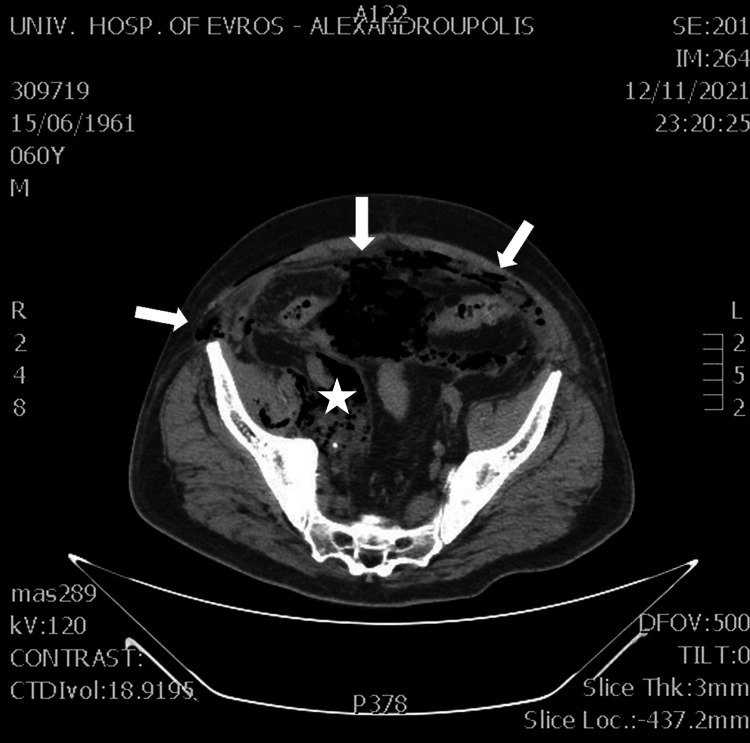
CT examination of the abdomen revealed free air in the peritoneal cavity (white arrows) and the right psoas muscle (white star). CT: computed tomography.

The patient was admitted to the internal medicine department and was administered broad-spectrum antibiotics and vasoconstrictor agents. However, a few hours later, the patient presented clinical deterioration with an acute abdomen, crepitus, and gray content in the bladder drainage. Because his clinical condition deteriorated, the surgical department was called for evaluation. After the clinical examination, an emergent computed tomography (CT) scan was recommended and performed. The CT scan revealed bubbles of free air in the abdominal wall (Figure 1), peritoneal cavity, and right psoas muscle (Figure 2), raising the suspicion of gangrenous cystitis.

**Figure 3:**
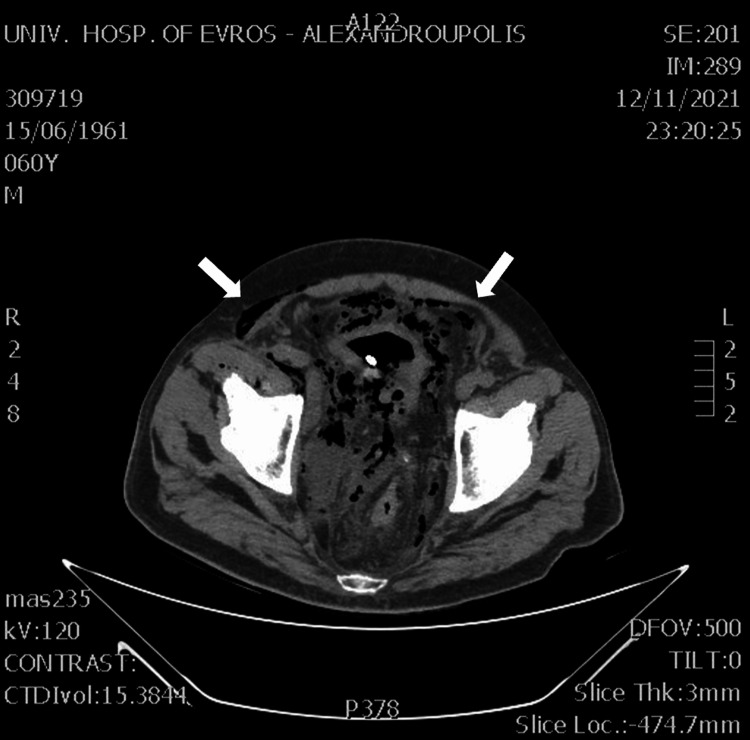
CT examination of the abdomen revealed fat stranding and free air around the bladder (white arrows). CT: computed tomography.

Additionally, fat stranding and free air around the bladder were observed (Figure 3), without any signs of organ perforation.

**Figure 4:**
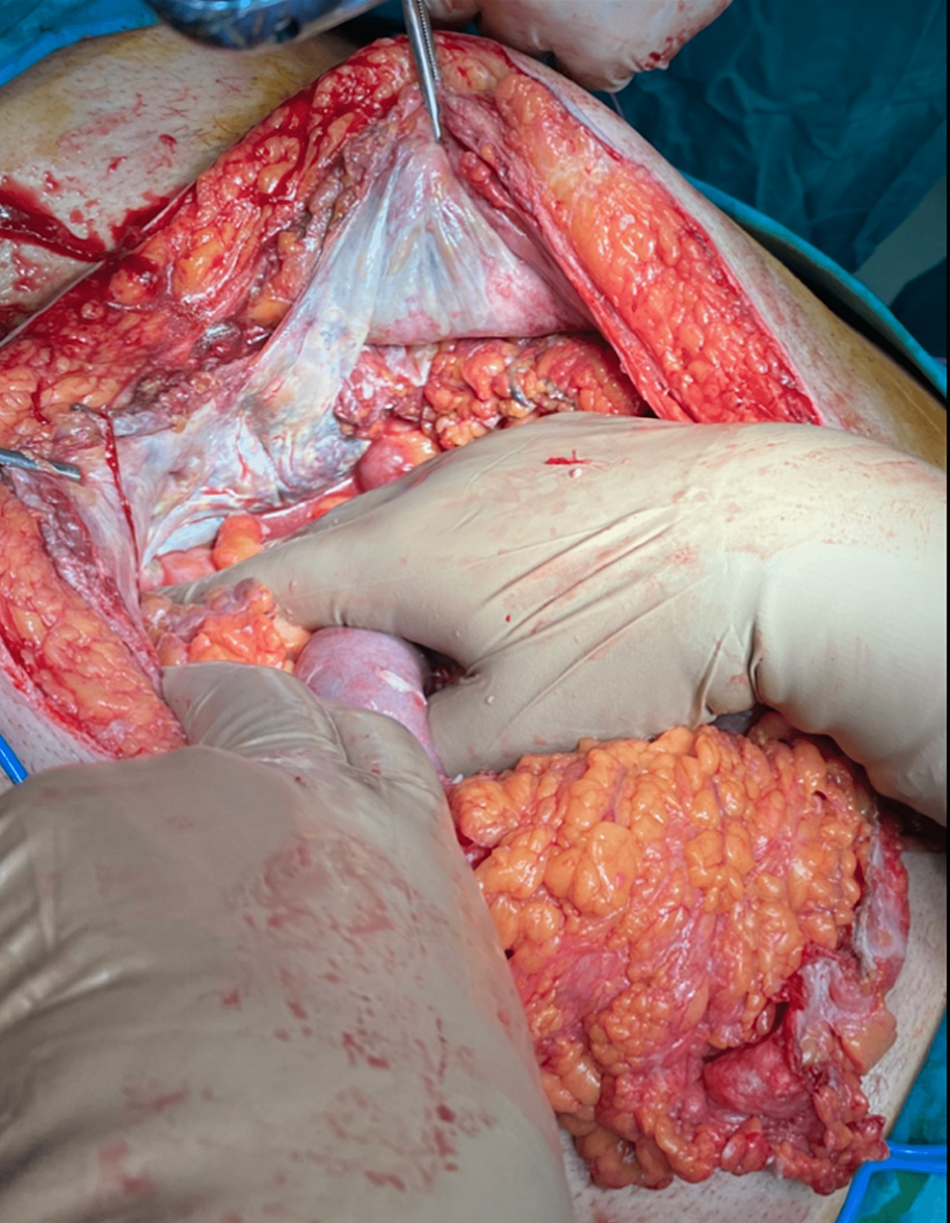
During laparotomy, extended peritoneal necrosis was observed.

**Figure 5:**
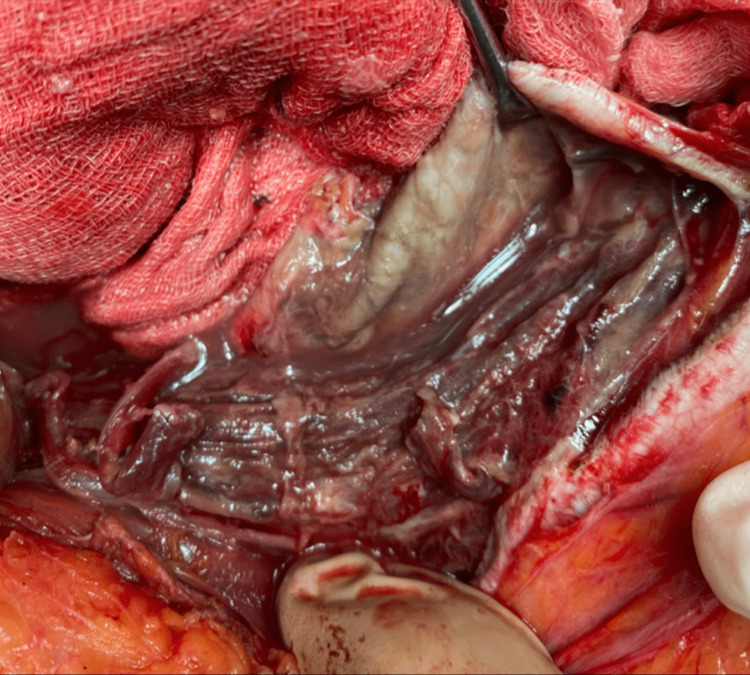
During laparotomy, abdominal wall necrosis was observed.

**Figure 6:**
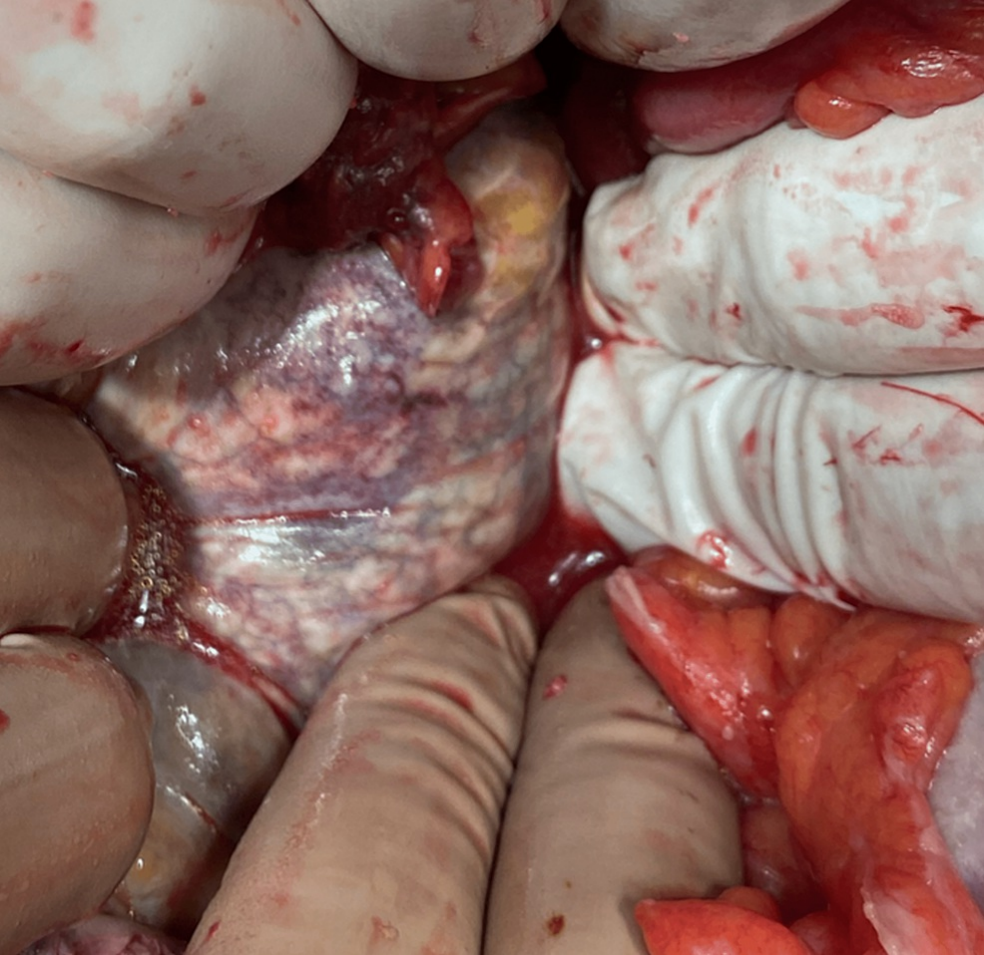
During laparotomy, urinary bladder necrosis was observed.

Both surgeons and urologists performed an emergency exploratory laparotomy. Excessive peritoneal (Figure 4), abdominal wall (Figure 5), and bladder necrosis (Figure 6) were observed intraoperatively.

Extensive debridement of necrotic tissues of the abdominal muscles, peritoneum, and bladder and surrounding tissues was initially performed. Intraoperative evaluation of the intestine distally from the Treitz ligament up to the rectum showed no relevant pathology. Moreover, owing to the generalized bladder necrosis, a radical cystectomy, prostatectomy, and bilateral ureterostomy were considered the appropriate approaches. Postoperatively, the patient presented hemodynamic instability and was transferred to the intensive care unit but unfortunately succumbed to cardiac arrest on the third postoperative day.

## Discussion

Gangrenous cystitis is a rare clinical condition, especially so since the widespread use of antibiotics [3], and it presents as necrosis of the urinary bladder mucosa and submucosa. When necrosis involves the entire bladder wall, it may lead to spontaneous rupture and acute peritonitis [4]. The prognosis remains poor, and the reported mortality is approximately 35% [5]. 

The etiological factors are prolonged labor (mainly in the pre-antibiotic era), malignant tumors in the pelvic area, urinary retention, vascular occlusion, topical chemotherapy, radiation, infection, and trauma. Most of the patients have comorbidities such as neurological illnesses and diabetes mellitus [3,6]. The symptoms are nonspecific, making the diagnosis extremely challenging. Typically, it may present with lower abdominal pain, dysuria, pyuria, hematuria, and urosepsis [1,3-5,7]. In delayed diagnosis, it may be considered as acute abdomen, resulting in a poor prognosis [5].

Imaging techniques such as ultrasound, CT, cystography, and cystoscopy contribute crucially to proper diagnosis. Of these, CT is the test of preference, but it is requested only in cases where signs and symptoms of acute abdomen are present [3-5,8]. In the present case, an emergent CT scan was performed. The CT scan showed bubbles of free air in the abdominal wall, peritoneal cavity, and right psoas muscle, which led to the diagnosis. 

If there is an early diagnosis, conservative treatment with the appropriate antibiotics, fluids, and bladder drainage may be successful. If the disease is caused by an anaerobic infection or the patient is unstable, surgical treatment should be immediate, and surgical debridement of necrotic tissue and drainage is the appropriate approach. The lifesaving treatment is radical cystectomy, but partial cystectomy may also be performed if the trigonum remains unaffected by the severe infection [1,3,5,6,8]. The initial surgical approach in our case was extensive debridement of necrotic tissue of the abdominal muscles, peritoneum, and bladder and surrounding tissues. Because of the generalized necrosis of the urinary bladder, the decision for radical cystectomy, prostatectomy, and bilateral ureterostomy was deemed appropriate. The delayed exploratory laparotomy and the aggressive surgical approach may have contributed to the unfortunate outcome.

## Conclusions

Gangrenous cystitis is a rare condition, and the clinical suspicion must be raised by the patient’s severe clinical condition as well as macroscopic examination of the urine. Finally, paraclinical control examinations should not delay the immediate surgical procedure, as the delay may aggravate the outcome.
